# A Lumped Parameter Method to Calculate the Effect of Internal Carotid Artery Occlusion on Anterior Cerebral Artery Pressure Waveform

**Published:** 2016-03-01

**Authors:** M. Abdi, M. Navidbakhsh, A. Razmkon

**Affiliations:** 1 School of Mechanical Engineering, Iran University of Science and Technology, Tehran, Iran; 2 Tissue Engineering and Biological Systems Research Laboratory, School of Mechanical Engineering, Iran University of Science and Technology, Tehran, Iran; 3 Department of Neurosurgery, Shiraz University of Medical Sciences, Shiraz, Iran

**Keywords:** Numerical modeling, Lumped method, Cardiovascular system, Internal carotid artery, Stenosis

## Abstract

**Background and Objective:**

Numerical modeling of biological structures would be very helpful tool to analyze hundreds of human body phenomena and also diseases diagnosis. One physiologic phenomenon is blood circulatory system and heart hemodynamic performance that can be simulated by utilizing lumped method. In this study, we can predict hemodynamic behavior of one artery of circulatory system (anterior cerebral artery) when disease such as internal carotid artery occlusion is occurred.

**Method:**

Pressure-flow simulation is one the leading common approaches for modeling of circulatory system behavior and forecasts of hemodynamic in numerous physiological conditions. In this paper, by using lumped model (electrical analogy), CV system is simulated in MATLAB software (SIMULINK environment).

**Results:**

The performance of healthy blood circulation and heart is modeled and the obtained results used for further analyses. The stenosis of internal carotid artery at different rates was, then, induced in the circuit and the effects are studied. In stenosis cases, the effects of internal carotid artery occlusion on  left anterior cerebral artery pressure waveform are investigated.

**Conclusion:**

The findings of this study may have implications not only for understanding the behavior of human biological system at healthy condition but also for diagnosis of diseases in circulatory and cardiovascular system of human body.

## Introduction

The most frequent causes of deaths in industrialized nations are cardiovascular diseases[1, 2]. Also, it is in the most productive age in which the people are affected by such diseases. The Circulatory system serves as a transporting vehicle for the nutrients, oxygen, carbon dioxide, hormones, blood cells and waste to and from the cells, as well as for the heat through blood convection. It consists of heart (with its four chambers: the right and left atrium and the right and left ventricle); systemic circulation (blood vessels in the body which carries oxygenated blood away from the heart to the body, and returns deoxygenated blood back to the heart); pulmonary circulation (blood vessels in the lungs which carry deoxygenated blood away from the heart, to the lungs, and returns oxygenated blood back to the heart); nervous and biochemical regulators (which modify the vessels’ parameters in order to regulate the blood flow rate and pressure in the organs in accordance with the actual demand)[3]. In this structure a variety of organs or mechanisms, such as baroreceptors, endocrine and nervous systems, respiration, metabolism, mental and physical exercise, etc., may influence as well. These mechanisms act in different time intervals. Even though the majority of the research regarding the physiological systems is based on experiments on animals, these have their limitations[4-6]. Understanding the difficult interactions and physiological functions of the CV system is not straightforward[2, 7, 8], but the use of recent technology is a helpful tool for this[6, 9, 10]. Recent development in mathematical skill, as well as more powerful and less expensive computers[1, 11, 12], has open greater possibilities for developing more sophisticated modeling methods of CV system (such as lumped method). An improved insight can be obtained by a structured analysis of the models which illustrate this system[13, 14], organizing the knowledge and the experimental observations in temporal and spatial order. In addition, a computer-based model of dynamic processes in the CV system could be applied in various tasks[5, 15]. 

The circulatory system can be considered as a complex hydraulic network where pulsatile pump simulates the heart. Different behavior can be observed at various locations of the closed loop. The 0D models (named equivalent electrical circuit models) regard as uniform the distribution of essential variables (blood pressure, volume and flow rate) in every isolated compartment (artery, vein, etc.) of the simulation, in every moment of time[1, 3, 9, 16]. 

The equivalent electrical circuit (lumped parameter models) that represent the main components of the system, (vasculature compartments and the heart with its valves), are suitable for the study of global distribution of the pressure, flow rate and blood volume, for specific physiological conditions. The variability of the parameters can be approximated by setting up the so called multi-compartments models, every compartment of which is regarded to be homogenous and expressed by a lumped parameter model[3, 5, 15-17]. 

The accurate measurement of the arterial pressure and flow is known to have great diagnostic and prognostic value and has inspired many attempts of simulation the circulatory system[2, 6, 10, 12, 14]. The major focus of study for a very perfect description of the CV system is undoubtedly the Navier–Stokes equations; however its enormous complexities make its practical implementation for the whole body impossible. While efforts have been made to combine it with the Windkessel model (WM) so as to attain both precise circulatory representation as well as reduction of the inherent complexities, the WM is extensively adopted in applications linking blood pressure, flow and heart load[4, 7, 8, 18]. Most researchers focus on parts of the circulatory system and in particular either the systemic or pulmonary arterial tree. The model suggested in this research, expands on these equations to cover the whole CV system where it is proposed an analogous variation of WM layout but without attempting to solve the resulting systems of equations. The importance of this closed loop approach is that it offers a functional insight in the complicated cardiovascular system in terms of directly providing estimates of the blood pressures, flows and volumes in various parts of the cardiovascular system.

In a nutshell, this paper includes the following steps:

a) The simplified block diagram of CV system is developed in new electrical circuit. 

b) The operation of a new circulatory system is simulated.

c) The pressure distribution of left anterior cerebral artery are assessed under normal and pathological (internal carotid artery stenosis) condition. 

## Materials and Methods

**Figure 1:**
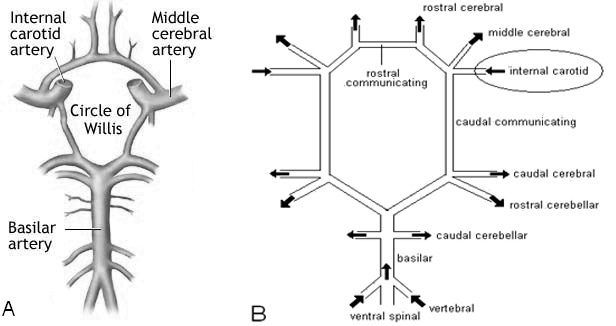
(A). Anatomic shape of circle of Willis. (B). This figure shows typical dynamic shape of circle of Willis.

The human cardiovascular system component consists of a pump, the heart, and an extensive system of tubes, the blood vessels that make up a closed, circular system. The vessels that leave the heart are called arteries, and those that return blood to the heart are called veins. Pumping of the heart generates an arterial blood pressure of about 100 mm Hg while venous blood returns to the heart at a pressure of about 5 to 10 mm Hg. The equivalent electrical circuit of circulatory system is based upon reference[1, 7, 11, 15]. Figure 1 shows the circle of Willis anatomically and dynamically. In electrical analogy, electric potential and current are corresponded to the average pressure and flow rate, respectively. A special vessel (or cluster of vessels) is characterized by means of its impedance, which is represented by a suitable combination of resistors, capacitors and inductors. The resistors are utilized to simulate viscous dissipation, while the capacitors account for vessel compliance; the ability to accumulate and release blood due to elastic deformation. Finally, the inductors are used to simulate inertia terms. Calculation of these parameters is according to previous studies[1, 4, 7, 9, 11, 15]. The diastolic filling period is assumed to last for 0.5 second and the systolic period for 0.3 second, for a heart period total of 0.8 second, corresponding to 60/0.8=75 beats/minute. 

In the case of internal carotid artery stenosis, the simulation is performed by changing electrical parameter and recalculating of electric lumped parameter such as resistance, inductance and compliance amount[1, 4]. By inserting these amounts into the computer simulation, the pressure (similar to voltage) wave propagations at ACA (anterior cerebral artery) are achieved.

We can derive the units of C, L and R:

C_units_=cm^4^*s^2^/gm

L_units_=gm/cm^4^

R_units_=gm/ (cm^4^*s)

Note that the viscosity µ of normal blood is µ=0.035 poise (gm/cm*s) and normal blood density is ρ=1.05 gm/cm^3^.pressure unit in this circuit is gm/cm*s^2^ (is not N/m^2^).but multiplying the pressure in mmHg unit by number 1332, the unit change to gm/cm*s^2^. 

## Results

**Figure 2:**
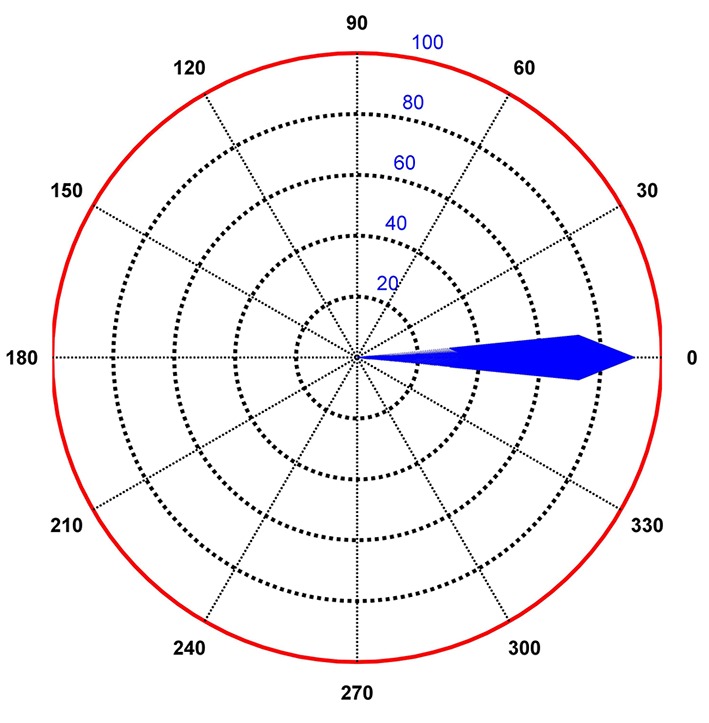
Left anterior cerebral artery pulse pressure under circumstance of healthy internal carotid artery (compass plot).

Figure 2 displays compass plot of left anterior cerebral artery of circle of Willis pressure pulse of complete circle of Willis. 

**Figure 3:**
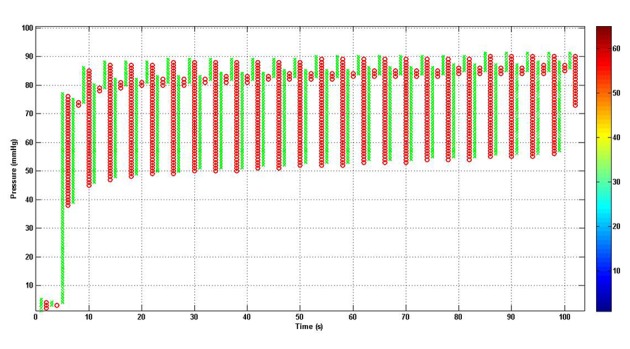
Left anterior cerebral artery pulse pressure under circumstance of healthy internal carotid artery (point figure).

**Figure 4:**
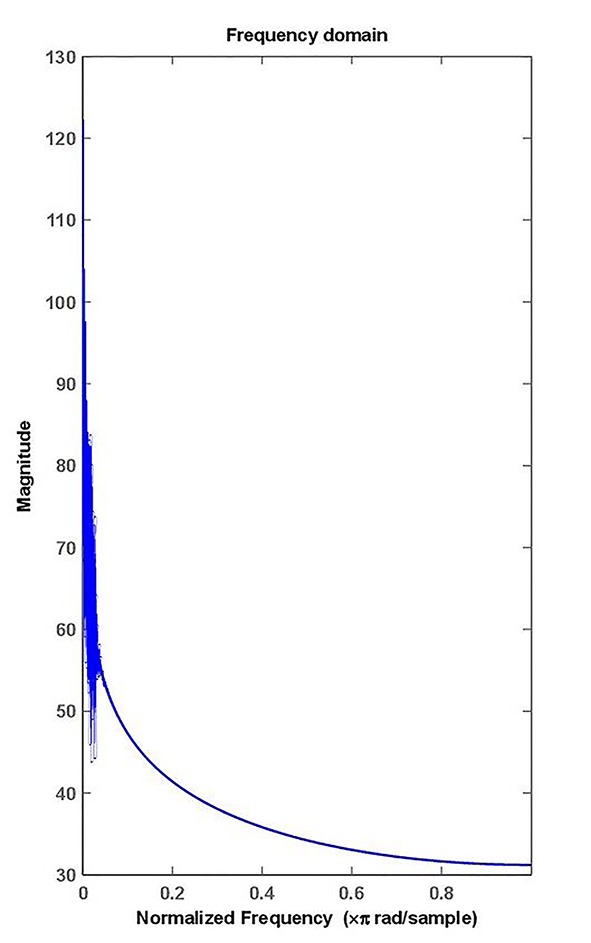
Left anterior cerebral artery pulse pressure under circumstance of healthy internal carotid artery (frequency plot).

**Figure 5:**
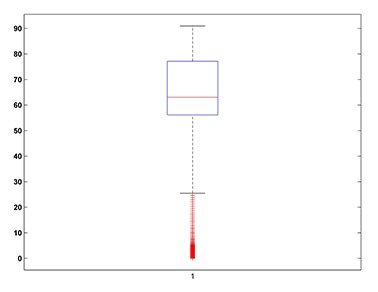
Left anterior cerebral artery pulse pressure under circumstance of healthy internal carotid artery (boxplot).

Difference between maximum and minimum amount of arterial pulse pressure varied from 62.5 mmHg to 96.1 mmHg for left anterior cerebral artery (Figure 2). Figures 3 to 5 show three major plot of anterior cerebral artery pulse pressure in the healthy internal carotid artery case. 

The pressure-time graph (point Figure) of left anterior cerebral artery is shown in Figure 3, where the waveform varies between 62.5 mmHg (volt) and 96.1mmHg (volt), respectively. The waveform starts from 62.5 mmHg and the peak is in 96.1 mmHg for healthy internal carotid. Our model is also able to predict frequency plot and boxplot (figures 4 and 5) the in vivo observed pressure patterns at left anterior cerebral artery. This graph shows that left anterior cerebral artery pressure varies between 62.5-96.1 mmHg (diastole-systole) for healthy internal carotid artery, respectively.

**Figure 6:**
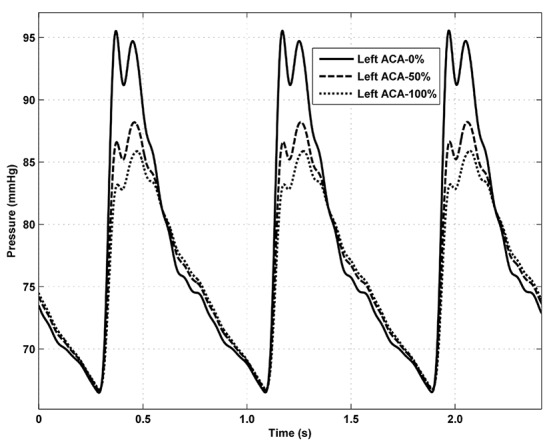
Left anterior cerebral artery pulse pressure as a function of occlusion’s growth rate in the healthy internal carotid artery case, in the 50% occlusion rate of internal carotid artery case and also under circumstance of complete occlusion of  internal carotid artery.

Finally, difference between maximum and minimum amount of arterial pulse pressure varied from 95 mmHg to 87 mmHg (corresponding to 50% and 100% occlusion rate of stenosis) for left anterior cerebral artery (Figure 6).

## Discussion

Under normal condition, each time period takes 0.8 second. This means the average heart rate for adult humans is 75 beats per minute in a normal undisturbed mode. A person’s blood pressure is usually expressed in terms of the systolic pressure over diastolic pressure (for left ventricle) and is measured in millimeters of mercury (mmHg), for example 140/50. Blood pressure usually ranges between 32 for the top or maximum number (systolic) and 5 for the bottom or minimum number (diastolic) for the right ventricle. The left and right ventricular pulse pressures are considered as entrance condition.

The results of the simulation performed based upon left and right ventricle pressure variations mimicking by controlled voltage source (voltage is equivalent blood pressure in electrical analogy method). 

There appears to be some decreased arterial pulse pressure through the left internal carotid artery when stenosis in this region is happened. Hence, the large occlusion rate of stenosis, the smaller efferent arteries pulse pressure (Figure 6) .moreover, the left anterior cerebral artery shows the uppermost drop rate in pressure (Figure 6). 

## Conclusion

This study had aim to model the pathological disorder in cardiovascular system using equivalent electrical system modeling. This paper would be considered as a very good approach in future studies in cardiovascular diseases field. The obtained results would have implications in the investigation of different diseases in cardiovascular diseases. 
